# Acute Effects of Forefoot Wedges on Concentric Power Output During Loaded Back Squats in Elite Female Handball Players: A Randomized Crossover Study

**DOI:** 10.3390/sports14080347

**Published:** 2026-08-11

**Authors:** Álvaro Gómez-del Pino, Laura Carrasco-Fernández, Javier Benítez-Porres, Gabriel Gijón-Noguerón, Joaquín Páez-Moguer, Manuel García-Sillero

**Affiliations:** 1 Department of Nursing and Podiatry, Faculty of Health Sciences, University of Málaga, 29071 Málaga, Spain; agdelpino@uma.es (Á.G.-d.P.); gagijon@uma.es (G.G.-N.); joaquinpaez@uma.es (J.P.-M.); 2 Department of Human Physiology, Physical Education and Sport, Faculty of Medicine, University of Málaga, 29010 Málaga, Spain; lauracarrasco.podologia@gmail.com (L.C.-F.); benitez@uma.es (J.B.-P.); 3 Internal Medicine UGC, Victoria Virgen University Hospital, Institute of Biomedical Research of Málaga (IBIMA), 29590 Málaga, Spain; 4 CIBER Physiopathology of Obesity and Nutrition (CIBEROBN), Carlos III Health Institute, 28029 Madrid, Spain; 5 Physiotherapy and Sport Department, Faculty of Biomedicine and Sports, University European of Andalucía, 29010 Málaga, Spain

**Keywords:** windlass mechanism, foot biomechanics, power output, explosive performance, forefoot wedge, biofeedback, handball, squat

## Abstract

The foot’s windlass mechanism (WM), engaged by hallux dorsiflexion, is a key determinant of lower-limb propulsive efficiency in explosive sports. This randomized within-subjects crossover study examined whether forefoot wedges of varying angulation, intended to facilitate the windlass mechanism, with or without real-time visual biofeedback, modify concentric power output during loaded back squats in elite female handball players. Seventeen professional athletes performed 3 × 3 back squats at 65% one-repetition maximum under six randomized conditions: three wedge angles (0°, 10°, and 30°) × biofeedback (present or absent). Peak concentric power (W) was the primary outcome; secondary outcomes included relative peak power (W·kg^−1^), concentric force (N), and concentric velocity (m·s^−1^), all recorded using dual force plates. The pre-specified two-way (3 × 2) repeated-measures ANOVA showed no significant main effects of wedge angle or biofeedback and no significant interaction for peak concentric power (all *p* > 0.05). A complementary exploratory Friedman analysis indicated an overall condition effect (χ^2^ = 14.01, *p* = 0.016; Kendall’s W = 0.165), with the highest descriptive values observed for the 30° wedge without biofeedback. However, pairwise comparisons did not remain significant after correction and did not isolate an independent wedge effect. Taken together, these findings are exploratory and hypothesis-generating and do not confirm that forefoot wedging acutely enhances power output in this population. Larger, pre-registered trials with direct biomechanical verification of windlass activation and longer-term performance outcomes are needed before specific wedge-based applications can be recommended in elite handball practice.

## 1. Introduction

An athlete’s ability to rapidly generate and transmit force to the ground is fundamental for explosive sports performance. In elite women’s handball, this demand is especially high during actions such as jumping, blocking, and cutting, which occur frequently each match. Indeed, ankle and forefoot injuries account for approximately 19% of all injuries in this sport [1]. In this context, the first-ray complex of the foot—particularly the windlass mechanism (WM)—is essential for effectively transmitting force from the calf muscles (triceps surae) to the ground, thereby optimizing lower-limb power output [2,3].

The WM refers to the coiling of the plantar fascia around the metatarsal head during dorsiflexion of the hallux (first metatarsophalangeal joint, 1MTPJ). This tightening of the plantar fascia elevates the medial longitudinal arch, induces rearfoot supination, and triggers external rotation of the tibia and femur [2,3]. In doing so, the WM transforms the foot into a rigid lever that minimizes energy dissipation and facilitates elastic energy storage and return during propulsion [4,5,6,7]. The efficiency of the WM is modulated by the strength and neuromuscular control of the intrinsic foot muscles, especially the abductor hallucis (AbH) and flexor hallucis brevis (FHB), as well as the long toe flexors [8,9]. For example, a larger cross-sectional area and greater voluntary activation of the AbH are associated with increased arch stiffness and reduced navicular drop in dynamic tasks [10,11]. Strengthening these muscles can enhance postural stability and performance in movements such as horizontal jumps and 90° changes in direction [12,13]. Moreover, toe-flexor strength shows moderate positive correlations with sprint acceleration, vertical jump height, and take-off dynamics [14,15]. High-load training of the toe flexors (e.g., “short-foot” or “doming” exercises) has been shown to increase arch stiffness and improve reactive jump performance indices [16,17].

In women’s handball, players perform between 30 and 40 jumps per match [1]. These athletes can generate peak power outputs of ~45–55 W·kg^−1^ during fast-break plays, where the ability to apply high vertical forces in ground contact times below 200 ms is critical for success [18,19]. The loaded squat exercise is a well-established test for evaluating vertical force output and the rate of force development (RFD) [20,21]. Notably, foot kinematics during a deep squat closely replicate those of the landing phase in jumping: as the hallux dorsiflexes, the WM tightens and the intrinsic foot flexors are engaged [6,22]. Therefore, force-platform assessment during squatting allows external force and power to be quantified throughout the eccentric and concentric phases of the movement [23,24]. These tools yield objective metrics—such as vertical impulse and peak power—that are invaluable for designing targeted strength programs and can be immediately utilized for athlete feedback [25,26].

Providing real-time biofeedback on variables like AbH–FHB muscle activation, plantar pressure distribution, or center-of-pressure trajectory has improved motor learning and jump technique in previous studies and can reduce chronic ankle instability rates [25,26]. In athletic populations, incorporating immediate feedback during foot-strengthening drills or squats has been linked to additional gains in intrinsic foot muscle strength (~12–15% increases) and improvements in knee/hip kinematics [27,28,29,30].

Previous research has also explored using rigid forefoot wedges to facilitate WM activation. In particular, different wedge treatments under the MTPJ (sometimes termed a “kinetic wedge”) have shown benefits for forefoot strength and mobility [31]. However, the acute effects of using different wedge angles—especially in combination with biofeedback—on neuromuscular performance remain largely unknown [32,33]. Notably, Tourillon et al. recently demonstrated improvements in propulsion kinetics following forefoot strengthening in athletes; however, most previous investigations have focused on chronic forefoot strengthening protocols or on kinetic wedges in unloaded or low-intensity tasks, and virtually no data exist on the acute effects of different forefoot wedge angles on concentric power output during heavy squats in elite female handball players [15]. Moreover, it remains unclear whether adding real-time visual feedback on force production provides any additional acute performance benefit beyond the mechanical facilitation of the windlass mechanism.

Therefore, the primary aim of this study was to examine the acute effects of forefoot wedges of varying inclination (0°, 10°, 30°), intended to facilitate the windlass mechanism, on peak concentric power output during loaded squats in elite female handball players. Secondary aims were to determine whether (i) forefoot wedging also affects concentric force and movement velocity, and (ii) the combined visual-feedback and verbal-encouragement condition provides any additional acute performance effect. Based on prior biomechanical literature linking forefoot dorsiflexion to windlass mechanism engagement, we hypothesized that squatting with a 30° forefoot wedge would be associated with higher peak concentric power output compared with a 0° control condition, with intermediate values expected at 10°. This dose–response hypothesis was derived from wedge geometry and previous kinematic studies of hallux dorsiflexion under load, rather than from direct measurement of windlass mechanism activation in the present sample. In addition, we explored whether the combined feedback condition produced additional acute effects relative to the no-biofeedback condition.

## 2. Materials and Methods

This study employed a prospective, randomized within-subject crossover design. Ethical approval was obtained prior to data collection from the Ethics Committee for Experimentation of the University of Malaga (CEUMA, Protocol No. 38-2019-H, approved in 2019). An internal study protocol and statistical analysis plan were developed and fixed as part of the corresponding doctoral project before testing began. Data collection took place in August 2022 following this pre-specified protocol. Formal trial registration in ISRCTN (ISRCTN33909124) was completed retrospectively on 12 June 2026 for administrative reasons related to doctoral programme requirements and did not involve any post hoc modification of outcomes or the analysis plan.

**Figure 1:**
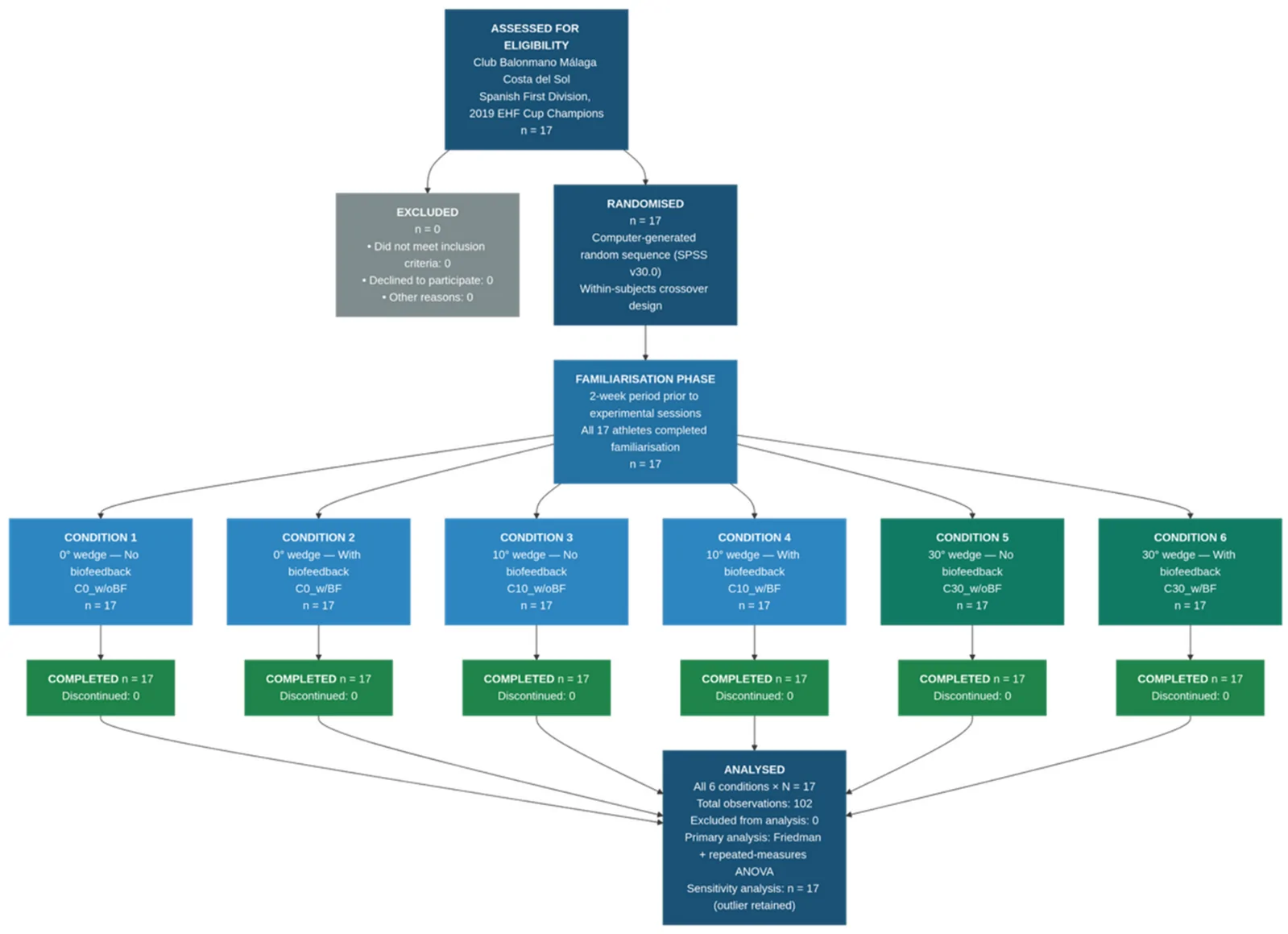
CONSORT 2025 flow diagram of the randomized within-subjects crossover trial [34]. All 17 eligible athletes were enrolled, randomized, and completed all six experimental conditions without dropout or missing data. Abbreviations: BF = real-time visual biofeedback; C0 = 0° forefoot wedge (flat control); C10 = 10° forefoot wedge; C30 = 30° forefoot wedge; w/BF = with biofeedback; w/oBF = without biofeedback.

The order of the six experimental conditions (C0 w/oBF, C0 w/BF, C10 w/oBF, C10 w/BF, C30 w/oBF, and C30 w/BF) was structured according to a six-sequence Williams design (Figure 1). Research Randomizer (version 4.0; Social Psychology Network, Middletown, CT, USA) was used to generate a complete six-condition sequence for each participant. Allocation concealment was implemented using six sequentially numbered, sealed opaque envelopes per participant (102 envelopes in total: 17 participants × 6 sessions). One envelope was opened immediately before each experimental session. Each participant completed one condition per session, with at least 48 h between consecutive sessions.

The primary outcome was peak concentric power (W) during the concentric phase of the squat, as it integrates both force and velocity components and represents the most performance-relevant variable for explosive actions in handball. Secondary outcomes included: (i) peak relative concentric power (W·kg^−1^); (ii) peak concentric velocity (m·s^−1^); (iii) peak and mean concentric force (N); and (iv) mean concentric power (W) and mean concentric velocity (m·s^−1^). All outcomes were pre-specified in the study protocol before data analysis.

### 2.1. Participants

Seventeen professional female handball players (age 29.9 ± 4.2 years, height 1.70 ± 0.07 m, body mass 67.2 ± 8.9 kg, and body mass index (BMI) 23.0 ± 1.7 kg·m^−2^) from Club Balonmano Málaga Costa del Sol (Spanish First Division, 2019 EHF Cup Champions) volunteered for this study.

Sample size was estimated a priori using G*Power v3.1 (University of Kiel, Kiel, Germany), assuming a medium within-subject effect size (f = 0.25), a nonsphericity correction ε = 1, and a correlation among repeated measures r = 0.5 for an omnibus repeated-measures comparison of the six conditions. Under these assumptions, 17 participants were required to achieve >80% power at α = 0.05. This calculation targeted the six-condition comparison and did not explicitly model the 3 × 2 factorial structure (wedge angle × biofeedback) later used as the confirmatory analysis. In the actual ANOVA, the observed effect sizes for peak concentric power were small (ηp^2^ ≈ 0.075, 0.012, 0.014 for wedge angle, biofeedback, and their interaction), indicating limited power for the factorial terms.

Inclusion criteria required a negative Jack’s test at the first metatarsophalangeal joint, confirming the absence of functional hallux limitus [35]. This clinical test verifies qualitative hallux mobility but does not quantify hallux dorsiflexion range of motion, medial arch height or stiffness, overall foot posture, or intrinsic foot muscle strength. These variables were not measured in the present study. Consequently, inter-individual variability in response to the wedge conditions cannot be mechanistically attributed to specific foot structural or strength characteristics. The absence of detailed foot profiling is acknowledged as a limitation.

All participants completed a two-week familiarization period with the squat protocol and testing equipment prior to experimental sessions.

### 2.2. Procedures

Initial meetings were held with the club’s technical staff to explain the experimental protocol and its logistical requirements. All participants received detailed information on the study procedures and provided written informed consent before beginning the tests.

**Figure 2:**
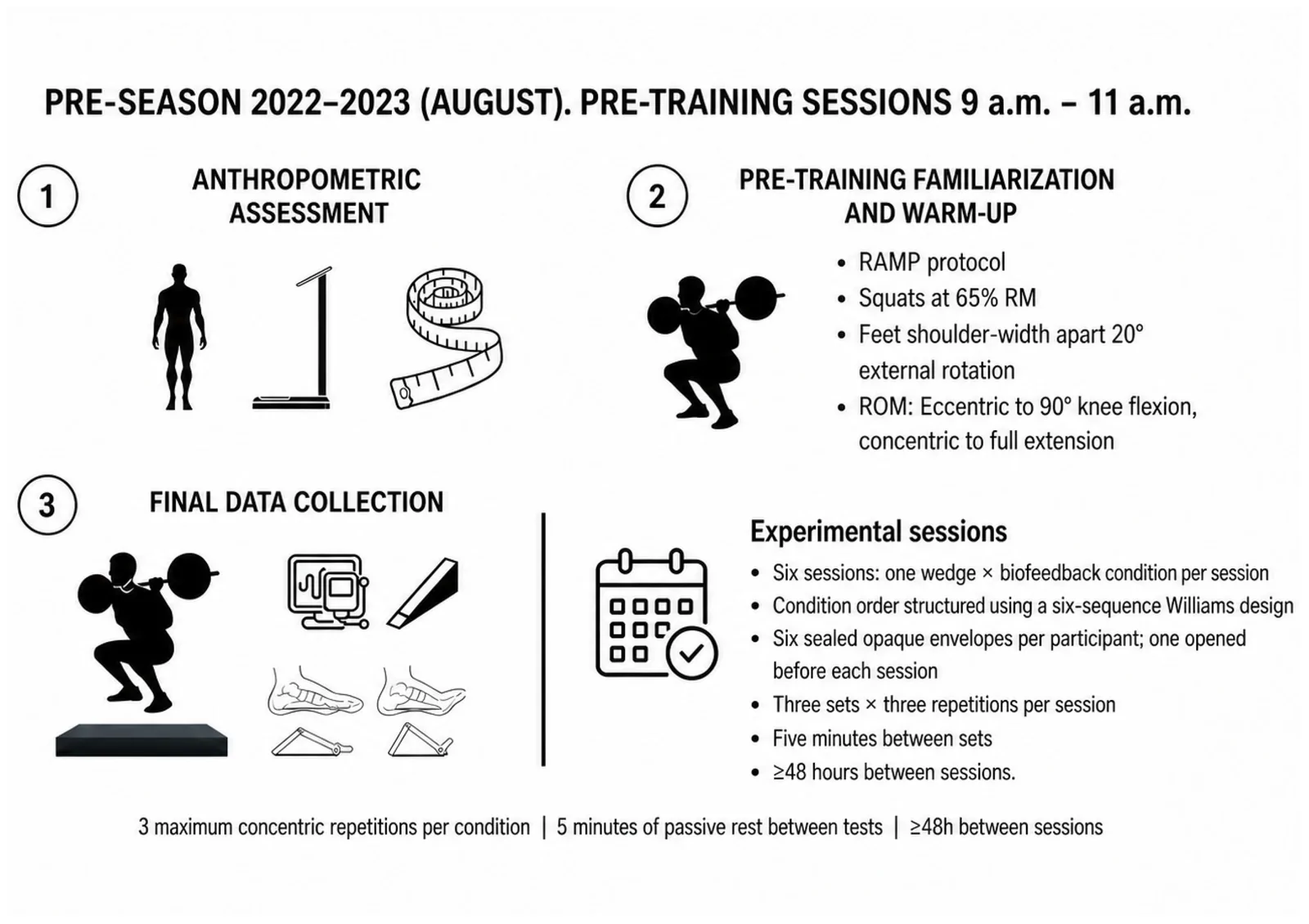
Organization of data collection across the study. A two-week familiarization period preceded experimental testing. Each participant subsequently completed the 1RM assessment and six experimental sessions, with one wedge × biofeedback condition per session and at least 48 h between consecutive sessions.

All assessments were carried out in the Exercise Physiology and Sports Medicine Laboratory at the University of Málaga. Testing took place during the pre-competitive phase of the season (August 2022) in morning sessions (09:00–11:00), before the day’s regular training. Participants were instructed to refrain from intense physical exercise for at least 12 h prior to each testing session to minimize fatigue effects. The overall data collection schedule and flow of sessions are illustrated in Figure 2.

### 2.3. Data Collection Techniques and Instruments

#### 2.3.1. Anthropometric Measurements

Height and body mass were measured using a wall-mounted stadiometer (Holtain Ltd., Crymych, UK) and a calibrated digital scale (SECA GmbH, Hamburg, Germany), respectively. BMI was then calculated. Body composition—including lean mass, fat mass, and muscle and fat percentage—was assessed by bioelectrical impedance analysis (InBody 770 device, InBody Co., Ltd., Seoul, Republic of Korea) according to manufacturer guidelines.

#### 2.3.2. Warm-Up and Familiarization

Each session began with a standardized warm-up following the RAMP protocol (Raise, Activate, Mobilize, Potentiate), including progressive squats on the Smith machine. This warm-up, including exercise selection, volume, and load progression, was identical across all six experimental sessions for every athlete. After the warm-up, each athlete performed several practice squat repetitions at approximately 65% of her estimated 1RM to reinforce technique and familiarity with the testing load. Practice repetitions were followed by a standardized rest interval before the experimental sets, in order to minimize any acute potentiation or fatigue effects on the first tested condition within each session.

#### 2.3.3. Maximal Strength Testing (1RM Squat)

The one-repetition maximum (1RM) back squat test was conducted in a separate session, at least 48 h before the first experimental trial for each athlete. This session followed the same general warm-up and squat technique practice described above but did not include any wedge or biofeedback conditions. The six experimental sessions (one condition per session) were scheduled only after the 1RM load had been established, with at least 48 h between the 1RM session and the first experimental session, to minimize residual fatigue effects on subsequent power measurements. In that session, each athlete first completed the general warm-up and technique practice described above. Every player first performed three sets of five squats at 20 kg, 30 kg, and 40 kg as incremental warm-ups. Then, using the same Smith machine apparatus, the load was progressively increased in smaller increments until the participant could no longer complete a full back squat (to 90° knee flexion) without assistance. The heaviest successfully lifted load was recorded as the 1RM. Squat depth was standardized to a 90° knee joint angle for all attempts, confirmed using a digital goniometer (Halo Medical Devices, Subiaco, Australia) and a height-adjustable bench placed beneath the athlete as a depth marker to ensure consistent squat depth for each athlete [36]. The protocol typically comprised three attempts at lighter loads, two at moderate loads, and one near-maximal attempt. Rest periods of ~3 min were given between submaximal attempts and 5 min before each maximal attempt, in line with recommended powerlifting protocols.

#### 2.3.4. Experimental Protocol

Following determination of each athlete’s 1RM, participants completed six experimental sessions at 65% of 1RM, one for each wedge × biofeedback condition. The order of the six conditions followed the participant-specific Williams sequence generated before testing. During each session, the assigned wedge angle and biofeedback status remained fixed, and the athlete performed three sets of three back squat repetitions. Five minutes of passive rest separated the sets, and at least 48 h separated consecutive experimental sessions.

All squat trials were performed on the Smith machine to maintain a consistent bar path and reduce balance demands. The athlete started each rep from an upright position (knees and hips fully extended). Foot placement was standardized to approximately shoulder-width apart with approximately 20° of external rotation, adjusted to each athlete’s natural stance.

For the wedged conditions, custom wedge supports were positioned bilaterally beneath the athletes’ forefeet (specifically under the head of the first metatarsal and hallux) during the trials. All tests were performed barefoot on the dual force-plate system to avoid the confounding influence of footwear on forefoot mechanics and wedge–foot interface. While the use of a Smith machine and barefoot squatting improved internal validity and measurement reliability, but reduced ecological validity and measurement reliability, it reduced ecological validity with respect to on-court handball movements and in-shoe conditions, as discussed later.

Three wedge angles were tested: 0° (flat control), 10°, and 30°. Custom wedges were fabricated using CAD/CAM technology (Autodesk Fusion, version 2.0.12200; Autodesk, Inc., San Rafael, CA, USA), from high-density EVA (Shore A 55–60). The 10° wedge measured 8 cm (length) × 2 cm (width) × 1.41 cm (apex height), and the 30° wedge measured 8 cm × 2 cm × 4.62 cm, corresponding to nominal inclinations of 10° and 30°, respectively, as confirmed by the CAD design and trigonometric calculation (tan^−1^[height/length]).

Although the wedge angle was fixed at 10° and 30°, actual hallux dorsiflexion achieved during dynamic squats may vary inter-individually due to foot anatomy, arch height, and load distribution. This represents a limitation of the current study. When placed under the forefoot (hallux region), the wedges were mechanically designed to induce dorsiflexion at the 1st metatarsophalangeal (MTP) joint, in line with prior descriptions of windlass mechanism engagement through hallux dorsiflexion [30]. The 10° wedge was selected to approximate dorsiflexion angles reported during the propulsive phase of gait, whereas the 30° wedge was intended to approach higher dorsiflexion angles under load. However, hallux dorsiflexion, plantar fascia tension, arch height/stiffness, and MTP kinematics were not directly measured in the present study; therefore, windlass mechanism activation is inferred based on device geometry and previous biomechanical work rather than verified in this sample.

During each squat repetition, athletes performed a controlled descent (eccentric phase) until reaching ~90° of knee flexion (thighs parallel to the floor), followed immediately by a maximal-effort concentric phase to return to full extension. Participants were instructed to exert maximum voluntary effort during the concentric phase of each repetition, keeping both feet fully supported on the platforms. Assessment staff visually monitored heel lift. Only minimal heel lift was permitted at the end of the concentric phase, after the main force-generating portion of the movement, to avoid restricting natural terminal extension. Any repetition exhibiting heel lift during the primary concentric phase (i.e., before reaching maximum force and power) was excluded from the analysis and immediately repeated with corrected technique.

In the biofeedback conditions, athletes received real-time visual feedback of their force–time curve on a 24-inch monitor (S24C310 model; Samsung Electronics, Suwon, South Korea) positioned 2 m in front of them at eye level and connected to the force platform software (VALD hub v2022.4, VALD Performance, Brisbane, QLD, Australia) during each repetition [24,33]. The monitor displayed: (i) a continuous force-time curve updated in real time (sampling rate: 1000 Hz) during squat execution; (ii) instantaneous force values (N) displayed numerically in large font (size: 48 pt) in the upper-right corner of the screen; and (iii) a horizontal reference line indicating the participant’s previous best peak force from the familiarization session, serving as a performance target. The display presented force rather than power because force is more directly interpretable as an acute feedback signal for athletes. Power output, being a derived variable (force × velocity), is less intuitively actionable as an acute feedback target. This choice is acknowledged as a potential limitation, discussed further.

Participants were instructed to: “Push as explosively as possible while watching the screen. Try to make the force curve steeper and the peak number higher than the reference line.” Verbal encouragement was provided during biofeedback trials using standardized phrases: “Push!”, “Harder!”, “Explode!” In non-biofeedback conditions, participants received only neutral instructions (“Execute the squat as trained”), the monitor was turned away, and no verbal encouragement or visual feedback was provided. Thus, biofeedback trials combined visual feedback with standardized verbal encouragement, whereas non-biofeedback trials included neither. This intentional asymmetry was chosen to maximize ecological validity for applied performance settings but introduces a motivational confound, as acknowledged in the Limitations section. Each experimental session corresponded to one of the six combinations of wedge angle and biofeedback, according to the participant-specific Williams sequence. Wedge angle and biofeedback status remained fixed throughout all three sets within a given session and were not randomized within sessions. All trials were recorded, and force–time data were analyzed post hoc by an investigator blinded to condition allocation.

#### 2.3.5. Force Plates

A dual force-plate system (ForceDecks, VALD Performance, Brisbane, QLD, Australia) recorded bilateral vertical ground reaction forces at 1000 Hz, synchronized across both plates. Signal processing used the manufacturer’s default filtering and phase-detection settings. The ForceDecks system has previously been validated against laboratory-grade piezoelectric force plates, with errors below 1% for peak force and derived kinetic variables. Movement onset was defined as a 20 N deviation from system weight. The concentric phase was defined from the point of zero vertical velocity at the bottom of the squat to the return of vertical force to system weight. Net vertical acceleration was computed as (F − body weight)/mass, and velocity was obtained by numerical integration of acceleration over time. Instantaneous power was calculated as force × velocity. Each experimental condition comprised three sets of three repetitions, resulting in nine repetitions per participant and condition. For peak outcomes, peak concentric force, peak concentric velocity, and peak concentric power were defined as the single highest repetition-level value recorded across the nine repetitions. For mean outcomes, mean concentric force, mean concentric velocity, and mean concentric power were calculated as the arithmetic mean of the corresponding repetition-level values across all nine repetitions. The three sets were not averaged separately. The resulting single condition-level value for each outcome and participant was entered into the statistical analyses.

## 3. Statistical Analysis

All statistical analyses were conducted using IBM SPSS Statistics v30.0 (IBM Corp., Armonk, NY, USA). Data normality was assessed with the Shapiro–Wilk test and visual inspection of Q–Q plots; sphericity was checked using Mauchly’s test, with Greenhouse–Geisser corrections applied when violated. Statistical significance was set at α = 0.05. Given the moderate sample size (*n* = 17) and the applied nature of the study, effect sizes (partial eta-squared [ηp^2^] and r) are reported alongside *p* values to aid interpretation of effect magnitude.

The pre-specified primary confirmatory analysis was the two-way repeated-measures ANOVA (3 × 2). The Friedman test was planned a priori as a complementary sensitivity analysis in the event of normality violations. Given that normality assumptions were not met for the primary outcome and selected secondary outcomes, the Friedman test results are presented alongside the ANOVA; however, the ANOVA framework remains the primary inferential structure, and Friedman results are interpreted as complementary sensitivity findings.

The analysis for the main outcome (peak concentric power, W) and all secondary outcomes was a two-way repeated-measures ANOVA with factors Wedge Angle (3 levels: 0°, 10°, 30°) and Biofeedback (2 levels: with, without). When a significant main effect or interaction was detected, Bonferroni-adjusted post hoc comparisons were performed. Partial eta-squared (ηp^2^) values were computed and interpreted as small (0.01), medium (0.06), and large (0.14).

For peak concentric power and secondary outcomes that did not meet the normality assumption, the pre-planned complementary Friedman analysis was conducted across the six experimental conditions. When an overall condition effect was identified, post hoc pairwise comparisons were performed using Wilcoxon signed-rank tests. The Holm–Bonferroni procedure was applied across the 12 pairwise comparisons conducted for each outcome to control the family-wise error rate. Statistical interpretation was based on adjusted *p* values; unadjusted *p* values were not used to infer specific between-condition differences.

In addition, linear and quadratic polynomial contrasts were performed to test for a progressive relationship between wedge angle (0° → 10° → 30°) and force/power production, independent of biofeedback. Individual responses were calculated as the participant-level difference in peak concentric power between C30 w/oBF and C0 w/oBF. The smallest worthwhile change threshold for peak power was set at 60 W, corresponding to 0.2 × the between-participant standard deviation. Participants were descriptively classified as responders when Δ exceeded +60 W, as showing minimal change when Δ was within ±60 W, and as reducers when Δ was below −60 W. A post hoc sensitivity analysis was performed by repeating the six-condition Friedman test after excluding the participant with the largest negative response in the C30 w/oBF − C0 w/oBF contrast. This participant was excluded only from the sensitivity analysis and was retained in all primary analyses.

### Measurement Reliability and Smallest Worthwhile Change

Although formal test–retest reliability analysis was not conducted as part of this study protocol, previous validation studies using the same ForceDecks system (VALD Performance, Brisbane, Australia) have reported excellent reliability for peak force (ICC > 0.95; CV = 3–5%) and peak power (ICC > 0.90; CV = 5–8%) during loaded squats [32].

The smallest worthwhile change (SWC) for peak force and power was estimated as 0.2 × between-subject SD, consistent with recommendations for athlete monitoring [37]. Based on our sample SD, SWC for peak force was ~75 N and for peak power ~60 W. The descriptive difference of approximately 50 N between the 30° and 0° conditions was below the estimated 75 N SWC and should not be interpreted as practically meaningful.

## 4. Results

Seventeen athletes completed all conditions. Participant characteristics are summarized in Table 1: mean age 29.9 ± 4.2 years, height 1.70 ± 0.07 m, body mass 67.2 ± 8.9 kg, and BMI 23.0 ± 1.7 kg·m^−2^. The mean one-repetition maximum (1RM) back squat was 137.1 ± 19.1 kg. The testing load for all conditions was set to 65% of 1RM (89.1 ± 12.4 kg), and each athlete completed 3 sets of 3 repetitions per session (9 repetitions per condition). These descriptive data are summarized in Table 1.

### 4.1. Primary Outcome: Peak Concentric Power

The two-way repeated-measures ANOVA with factors Wedge Angle (0°, 10°, 30°) and Biofeedback (with, without) did not reveal a statistically significant Wedge × Biofeedback interaction for peak concentric power (F(2, 32) = 0.23, *p* = 0.794, ηp^2^ = 0.014). Main effects of Wedge Angle and Biofeedback are reported in Table 2 and are interpreted in light of the complementary non-parametric analyses and effect sizes.

Complementary Friedman sensitivity analyses indicated overall condition effects for absolute peak concentric power (χ^2^ = 14.01, *p* = 0.016) and relative peak power (χ^2^ = 12.61, *p* = 0.027). However, no post hoc pairwise comparison for either outcome remained statistically significant after Holm–Bonferroni adjustment. Descriptive statistics for peak concentric power and related concentric outcomes across all six conditions are presented in Table 3.

**Figure 3:**
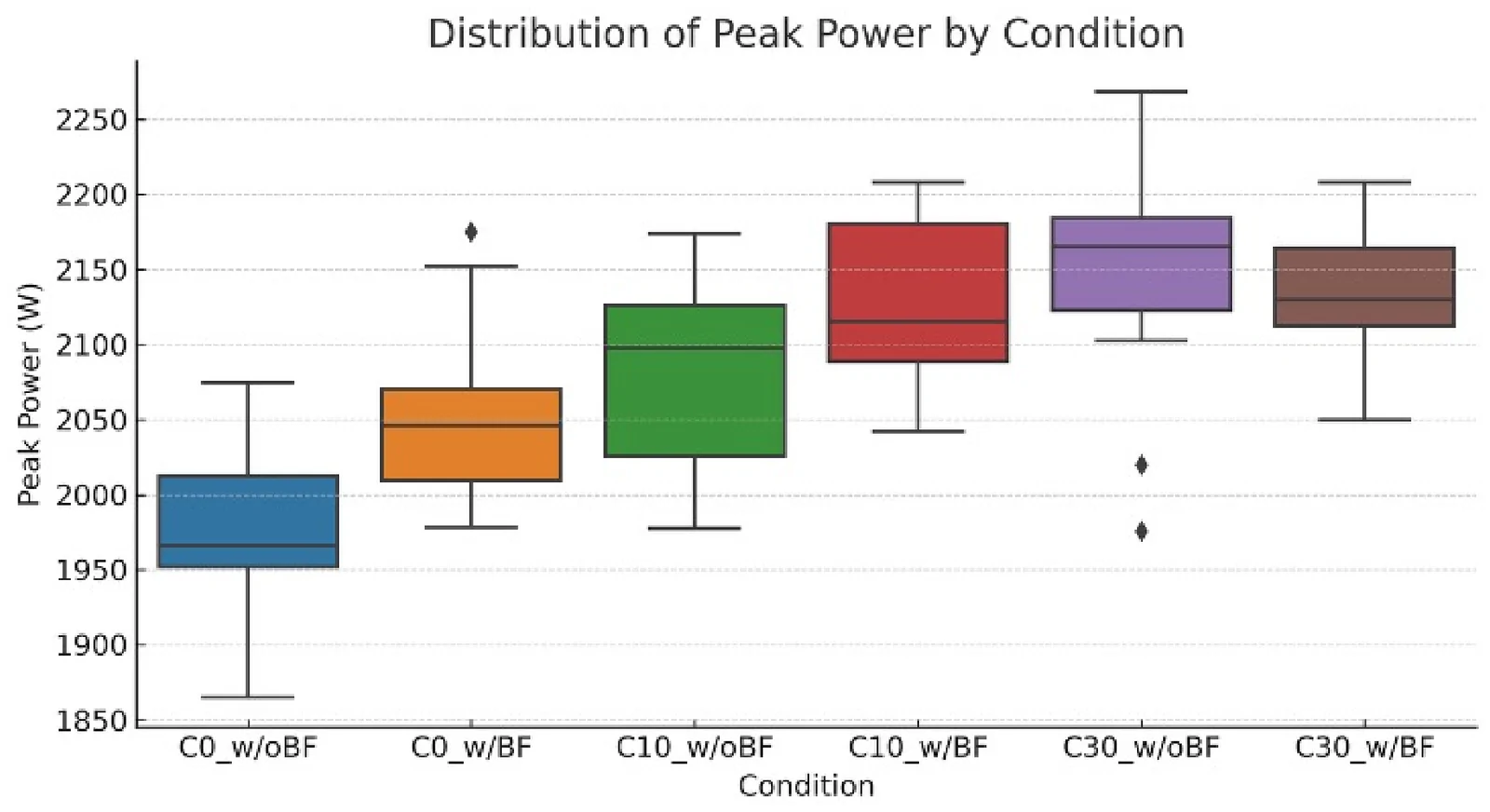
Distribution of peak concentric power (W) across the six experimental conditions (*n* = 17). Note: Each boxplot represents one condition: 0°, 10°, and 30° forefoot wedge × with or without real-time visual biofeedback. The central line indicates the median, ♦ indicates the mean, box edges represent the 25th and 75th percentiles, and whiskers represent 1.5 × IQR. The complementary Friedman analysis indicated an overall condition effect (χ^2^ = 14.01, *p* = 0.016); however, no post hoc pairwise comparison remained statistically significant after Holm–Bonferroni adjustment.

Among the six experimental conditions, the highest descriptive mean peak concentric power was observed in C30 w/oBF (2188.7 ± 328.9 W), whereas the lowest was observed in C0 w/BF (1994.5 ± 317.1 W). The complementary Friedman analysis indicated an overall condition effect (χ^2^ = 14.01, *p* = 0.016); however, none of the post hoc Wilcoxon pairwise comparisons remained statistically significant after Holm–Bonferroni adjustment. Therefore, the omnibus result indicates non-uniformity across the six conditions but does not identify a specific between-condition difference or an independent wedge effect (Figure 3).

### 4.2. Secondary Outcome: Concentric Force

Peak concentric force showed small descriptive differences across wedge angles. When values were collapsed across biofeedback conditions, descriptive mean peak force was higher at 10° and 30° than at 0°; however, the main effect of Wedge Angle did not reach statistical significance, and no pairwise comparison remained significant after Holm–Bonferroni adjustment. No significant main effect of Biofeedback or Wedge × Biofeedback interaction was observed. Accordingly, the present results do not identify a specific wedge angle or experimental condition associated with greater peak concentric force.

### 4.3. Secondary Outcome: Concentric Velocity

The complementary Friedman sensitivity analysis indicated an overall condition effect for maximal concentric velocity (χ^2^ = 12.32, *p* = 0.031). However, none of the post hoc Wilcoxon pairwise comparisons remained statistically significant after Holm–Bonferroni adjustment. The condition-specific differences were small and inconsistent, and the results do not identify a specific wedge or biofeedback condition associated with greater maximal concentric velocity.

### 4.4. Secondary Outcome: Biofeedback

In the 3 × 2 repeated-measures ANOVA, Biofeedback showed no significant main effect on peak concentric power, mean concentric power, relative peak power, or relative mean power. When values were collapsed across wedge angles at the participant level, the differences between biofeedback and no-biofeedback conditions were small and non-significant for all power outcomes (all *p* ≥ 0.513; ηp^2^ = 0.007–0.027; Table 4). Accordingly, the present data provide no evidence that the combined visual-feedback and verbal-encouragement condition acutely improved concentric power output.

### 4.5. Individual Response Variability

Individual responses were calculated as the difference in peak concentric power between C30 w/oBF and C0 w/oBF. Using an SWC threshold of 60 W, 12 of the 17 participants (70.6%) were descriptively classified as responders, no participant showed minimal change within ±60 W, and 5 participants (29.4%) were classified as reducers. Individual changes ranged from −1573 W to +1701 W.

**Figure 4:**
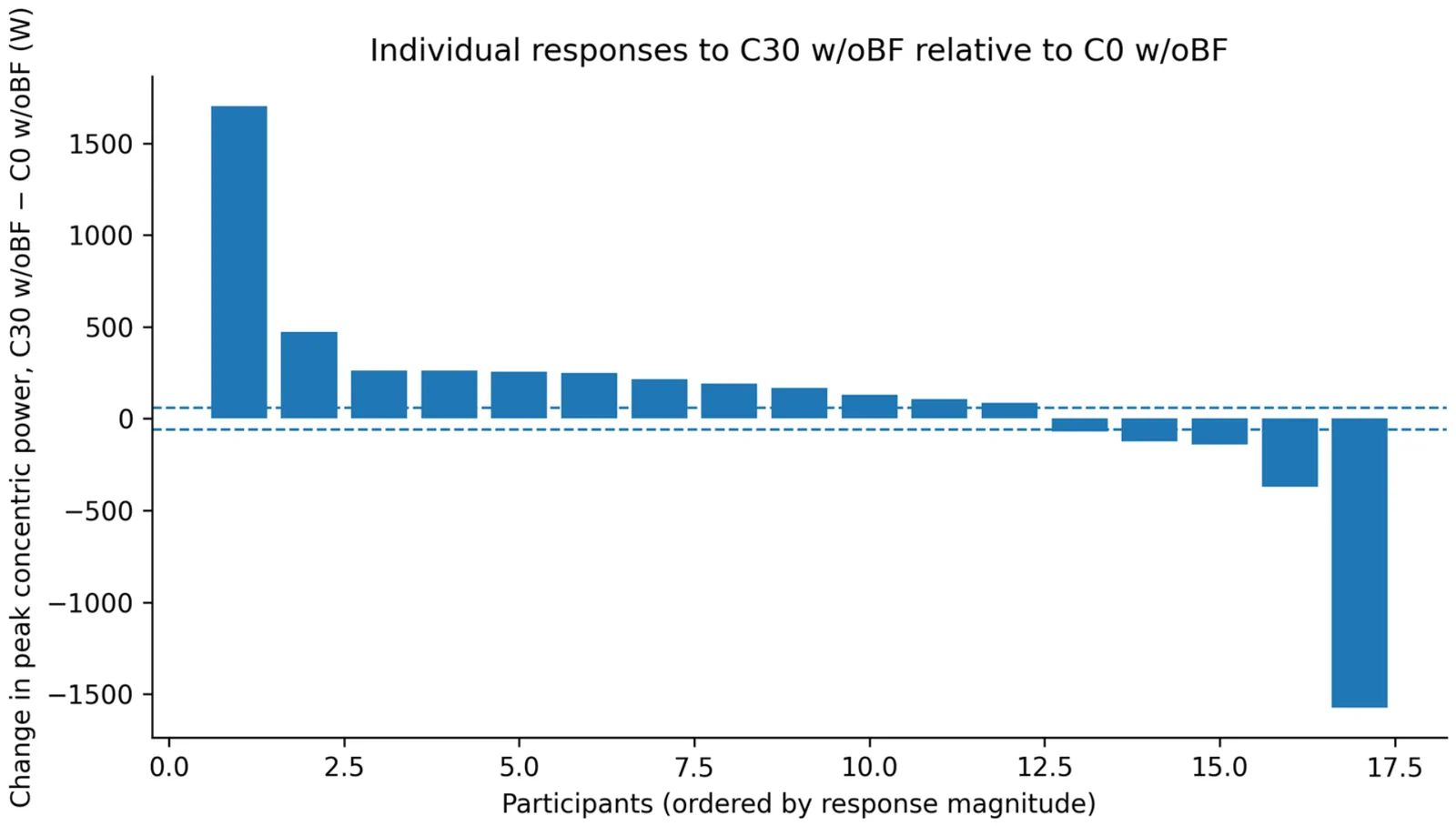
Individual changes in peak concentric power between the 30° wedge and control conditions without biofeedback (C30 w/oBF − C0 w/oBF; *n* = 17). Positive values indicate higher peak power in C30 w/oBF, whereas negative values indicate lower peak power. Dashed horizontal lines represent the smallest worthwhile change threshold (±60 W). Participants are ordered from the largest positive to the largest negative response.

The largest negative response was Δ = −1573 W. Verification of the original ForceDecks record confirmed that the underlying condition values were not attributable to a data-entry or processing error. A post hoc sensitivity analysis excluding this participant—equivalent to excluding six condition-level repeated-measures observations—yielded a comparable overall Friedman result (χ^2^ = 15.50, *p* = 0.008; *n* = 16), compared with χ^2^ = 14.01 and *p* = 0.016 in the complete sample (*n* = 17). After exclusion, individual changes ranged from −371 W to +1701 W, with 12 responders, no participants showing minimal change, and 4 reducers. The participant was therefore retained in all primary analyses (Figure 4).

The marked inter-individual variability in peak power responses to the wedge conditions may be influenced by unmeasured factors such as plantar fascia stiffness, medial arch compliance, first MTP joint mobility, and intrinsic foot muscle strength, which have been shown in other contexts to modulate windlass mechanics and explosive performance [30]. However, none of these variables were directly assessed in the present study, so any link between foot structural characteristics and wedge responsiveness must be regarded as speculative.

## 5. Discussion

### 5.1. Main Findings

To the best of the authors’ knowledge, this study is the first to systematically examine the acute effects of forefoot wedges of varying inclination, intended to facilitate the windlass mechanism, combined with real-time biofeedback, on concentric power output during loaded back squats in elite female handball players.

The pre-specified 3 × 2 repeated-measures ANOVA did not identify significant main effects of Wedge Angle or Biofeedback, or a significant Wedge × Biofeedback interaction, for peak concentric power. A complementary Friedman analysis indicated an overall condition effect (χ^2^ = 14.01, *p* = 0.016), suggesting that peak power was not uniformly distributed across the six experimental conditions. However, none of the post hoc pairwise comparisons remained statistically significant after Holm–Bonferroni adjustment. Although C30 w/oBF showed the highest descriptive mean, the analyses did not identify between-condition differences or establish an independent effect of wedge angle. Accordingly, the Friedman finding should be interpreted as exploratory and hypothesis-generating rather than as confirmatory evidence of a performance benefit.

Individual responses to C30 w/oBF relative to C0 w/oBF were highly heterogeneous, ranging from −1573 W to +1701 W. Based on the 60 W SWC threshold, 12 participants were descriptively classified as responders and 5 as reducers, with no participant showing a change within ±60 W. These classifications should be interpreted cautiously because they were based on a single acute assessment rather than repeated individual-level measurements.

Peak concentric force showed small descriptive differences across wedge angles, but the main effect of Wedge Angle was not statistically significant, and no adjusted pairwise comparison identified a specific between-condition difference. Maximal concentric velocity also showed no consistent condition-specific pattern. Therefore, these secondary outcomes do not provide independent evidence that the 30° wedge improves performance. Although some descriptive patterns may be compatible with proposed windlass-related mechanisms [30], hallux dorsiflexion, plantar fascia strain, medial longitudinal arch deformation or stiffness, and first metatarsophalangeal joint kinematics were not measured. Any mechanistic interpretation must therefore remain speculative.

The absolute power values observed in the present sample can be contextualized against vertical-jump performance data reported in professional female handball players [38], although differences in task, external load, and movement characteristics preclude direct numerical comparison. More broadly, previous research indicates that plant-foot kinetics, intrinsic foot muscle function, and windlass-related foot mechanics may contribute to propulsion and jumping or running performance [39,40,41,42,43]. Force-time analyses have also been used to characterize maximal jump and landing performance and to support the interpretation of force-platform-derived variables [44,45]. These studies provide biomechanical and methodological context for the present experiment, but they do not demonstrate that the 30° wedge improved performance in the current sample.

### 5.2. Effects of Biofeedback

In the biofeedback conditions, athletes received real-time visual feedback of their force–time curve and instantaneous force values on a monitor connected to the ForceDecks software, together with standardized verbal encouragement (‘Push!’, ‘Harder!’, ‘Explode!’) during each repetition. In the non-biofeedback conditions, participants received neutral instructions (‘Execute the squat as trained’) and no verbal encouragement or visual feedback. Accordingly, the Biofeedback factor in the 3 × 2 repeated-measures ANOVA represents a combined feedback–motivation condition versus a neutral control, and the present design does not allow isolation of the independent effect of visual feedback alone.

The 3 × 2 repeated-measures ANOVA showed no significant main effect of Biofeedback on any absolute or relative power outcome, and the corresponding effect sizes were small. Values collapsed across wedge angle were also slightly lower, rather than higher, in the biofeedback condition, although these differences were not statistically significant. Accordingly, the present findings do not support an acute ergogenic effect of the combined visual-feedback and verbal-encouragement condition. Because visual feedback and verbal encouragement were delivered together, their independent contributions cannot be determined. Motor-learning research indicates that responses to feedback may depend on attentional focus, task familiarity, feedback modality, and repeated practice [46,47]. However, these mechanisms were not directly assessed in the present study and should not be invoked as causal explanations for the absence of a null acute effect. Whether repeated exposure produces different effects remains unknown and should be evaluated in studies that standardize encouragement across experimental conditions.

### 5.3. Practical Implications

From an applied standpoint, the present acute findings should be considered hypothesis-generating rather than prescriptive. The non-significant primary ANOVA, the absence of statistically significant pairwise differences after correction, and the marked inter-individual heterogeneity preclude recommending routine use of 30° forefoot wedges in warm-ups or competition at this stage. Future adequately powered trials with direct biomechanical verification of windlass activation and longer-term performance outcomes are needed before specific wedge prescriptions can be formulated for elite handball practice.

## 6. Limitations

Several limitations should be considered when interpreting the present findings. First, the sample size was modest (*n* = 17) and consisted exclusively of elite female handball players from a single professional team. This homogeneous, highly trained cohort improves internal validity but limits generalizability to male athletes, younger or lower-level players, and other sports. Moreover, this was an acute trial: we examined only single-session responses to the wedge and biofeedback interventions. It remains unknown whether repeated exposure would produce different or sustained performance effects or neuromuscular adaptations.

Second, the study was designed primarily as a performance trial rather than as a mechanistic experiment. All squats were performed barefoot on a Smith machine, which improved standardization and measurement reliability but reduced ecological validity relative to free-bar squats or on-court movements performed in handball shoes. Windlass mechanism activation was inferred from wedge geometry and previous biomechanical literature rather than verified in the present analysis. Hallux dorsiflexion, plantar fascia strain, medial longitudinal arch deformation or stiffness, and first metatarsophalangeal joint kinematics were not quantified. Consequently, the present findings cannot establish the biomechanical pathway through which the wedge conditions may have influenced performance. Future studies incorporating synchronized foot kinematics, plantar fascia and arch measurements, and fully reported neuromuscular assessments are needed to evaluate the proposed mechanism.

Third, a six-sequence Williams design was used to structure the order of the experimental conditions, and consecutive sessions were separated by at least 48 h to reduce potential first-order carry-over and fatigue effects. Participant-specific condition sequences were documented in the allocation records; however, sequence and period variables were not incorporated into the final analytical dataset, and no formal analysis of period, sequence, or carry-over effects was performed. Consequently, residual influences related to progressive familiarization, cumulative fatigue, motivation, or session order cannot be excluded.

Finally, several potentially relevant sources of variability were not controlled. In this all-female sample, menstrual cycle phase was not recorded and could have introduced additional noise in neuromuscular performance measures. We also observed substantial inter-individual variability in the acute C30 w/oBF − C0 w/oBF contrast, with 12 of 17 participants descriptively classified as responders and 5 as reducers using the 60 W SWC threshold. These classifications were derived from a single acute assessment and should not be interpreted as evidence of reproducible individual responsiveness.

## 7. Conclusions

In this randomized within-subject crossover study, the pre-specified 3 × 2 repeated-measures ANOVA showed no significant main effects of Wedge Angle or Biofeedback and no significant Wedge Angle × Biofeedback interaction for peak concentric power during loaded back squats in elite female handball players. A complementary Friedman analysis indicated an overall condition effect, but no post hoc pairwise comparison remained statistically significant after Holm–Bonferroni adjustment. Therefore, the present data do not demonstrate that forefoot wedge angle or the combined visual-feedback and verbal-encouragement condition acutely modifies peak concentric power in this sample. Individual-response classifications were heterogeneous and were based on a single acute assessment. Larger, pre-registered studies incorporating direct biomechanical measurements and repeated individual-level testing are required to determine whether the observed condition-level variability is reproducible and performance-relevant.

## Figures and Tables

**Table 1:** Descriptive statistics of participants’ physical characteristics (*n* = 17). Values are mean ± SD.

Variable	Mean ± SD
Age (years)	29.88 ± 4.23
Height (m)	1.70 ± 0.07
Weight (kg)	67.21 ± 8.92
BMI	23.04 ± 1.72
Muscle Mass (kg)	29.71 ± 3.20
Fat Mass (kg)	14.42 ± 4.92
Body Fat (%)	20.83 ± 4.51
1RM (kg)	137.12 ± 19.09
65% 1RM (kg)	89.13 ± 12.41

**Table 2:** Two-way repeated-measures ANOVA for peak concentric power, with factors Wedge Angle and Biofeedback (*n* = 17).

Source	df	F	*p*	ηp^2^
Wedge Angle (0°, 10°, 30°)	2, 32	1.30	0.288	0.075
Biofeedback (with, without)	1, 16	0.20	0.663	0.012
Wedge × Biofeedback	2, 32	0.23	0.794	0.014

Note: Values correspond to a two-way repeated-measures ANOVA for peak concentric power with Wedge Angle (0°, 10°, 30°) and Biofeedback (with vs. without real-time visual feedback) as within-subject factors (*n* = 17). Partial eta-squared (ηp^2^) is reported as an effect-size measure for each effect. No main effects or interactions reached statistical significance at α = 0.05 in this ANOVA framework; complementary non-parametric analyses are reported in the main text.

**Table 3:** Descriptive statistics (mean ± SD) for concentric outcomes across the six experimental conditions (*n* = 17).

Variable	C0 w/oBF	C0 w/BF	C10 w/oBF	C10 w/BF	C30 w/oBF	C30 w/BF
Peak Power (W)	2081.3 ± 586.9	1994.5 ± 317.1	2116.2 ± 236.6	2123.7 ± 325.3	2188.7 ± 328.9	2166.4 ± 523.0
Mean Power (W)	949.9 ± 365.7	913.5 ± 251.8	980.1 ± 244.1	985.2 ± 283.8	1016.3 ± 307.2	1005.3 ± 354.0
Rel. Peak Power (W·kg^−1^)	30.7 ± 7.7	29.6 ± 4.3	31.6 ± 3.6	31.4 ± 3.6	32.5 ± 5.5	31.5 ± 5.9
Rel. Mean Power (W·kg^−1^)	13.3 ± 4.1	12.8 ± 1.8	13.8 ± 2.4	13.7 ± 2.0	14.2 ± 3.4	13.7 ± 2.9
Peak Force (N)	2150.9 ± 257.5	2162.5 ± 283.2	2187.6 ± 284.3	2198.9 ± 312.0	2197.4 ± 265.9	2215.1 ± 292.6
Mean Force (N)	1636.3 ± 174.9	1648.4 ± 195.0	1645.7 ± 183.4	1644.8 ± 191.9	1644.1 ± 165.3	1646.8 ± 172.2
Peak Velocity (m·s^−1^)	1.1 ± 0.3	1.2 ± 0.4	1.1 ± 0.2	1.1 ± 0.2	1.0 ± 0.1	1.1 ± 0.2
Mean Velocity (m·s^−1^)	0.6 ± 0.2	0.6 ± 0.2	0.6 ± 0.1	0.6 ± 0.1	0.6 ± 0.1	0.6 ± 0.1

Note: Values represent participant-level condition summaries calculated from nine repetitions, as described in the Methods, and are presented as group mean ± standard deviation (*n* = 17). Outcomes include peak and mean concentric power (absolute and relative), peak and mean concentric force, and peak and mean concentric velocity. Abbreviations: C0 = 0° forefoot wedge (no wedge); C10 = 10° wedge; C30 = 30° wedge; w/BF = with visual biofeedback; w/oBF = without biofeedback.

**Table 4:** Participant-level values collapsed across wedge angle and main effect of Biofeedback from the 3 × 2 repeated-measures ANOVA (*n* = 17).

Variable	Condition	Mean ± SD	Mean Difference (w/BF–w/oBF)	F	ρ-Value	η^2^_ρ_
Peak Power (W)	w/oBF vs w/BF	2128.75 ± 283.16 vs 2094.84 ± 326.46	−33.90	0.197	0.663	0.012
Mean Power (W)	w/oBF vs w/BF	982.11 ± 260.38 vs 968.00 ± 270.80	−14.11	0.115	0.739	0.007
Peak Rel. Power (W·kg^−1^)	w/oBF vs w/BF	31.61 ± 3.52 vs 30.82 ± 3.26	−0.79	0.447	0.513	0.027
Mean Rel. Power (W·kg^−1^)	w/oBF vs w/BF	13.79 ± 2.14 vs 13.41 ± 1.25	−0.39	0.375	0.549	0.023

Note: Values are presented as mean ± standard deviation. For each participant, outcomes were first averaged across the three wedge angles separately for the no-biofeedback condition (C0 w/oBF, C10 w/oBF, and C30 w/oBF) and the biofeedback condition (C0 w/BF, C10 w/BF, and C30 w/BF). The reported difference was calculated as w/BF minus w/oBF. F, *p*, and partial eta-squared values correspond to the main effect of Biofeedback in the 3 × 2 repeated-measures ANOVA. No significant main effect of Biofeedback was observed for any power outcome.

## Data Availability

The datasets presented in this article are not readily available because of contractual agreements with the club. Requests to access the datasets should be directed to the corresponding author.
